# Telemedicine Versus In-Person Visits in Postoperative Care in Orthopedic Patients: Follow-Up Study From North India

**DOI:** 10.7759/cureus.18399

**Published:** 2021-09-30

**Authors:** Kuldip S Sandhu, Akashdeep Singh, Akashdeep Singh, Daljinder Singh, Annie Sandhu

**Affiliations:** 1 Orthopedics, Government Medical College, Patiala, IND; 2 Emergency Department, All India Institute of Medical Sciences, Rishikesh, IND

**Keywords:** follow up, orthopedic patients, postoperative care, teleconsultation, telemedicine

## Abstract

Background: Telemedicine is improving healthcare delivery in orthopedic patients but the data regarding this are scarce from India, especially North India.

Methodology: During this one-year prospective observational study, all patients with fractures of the upper end of tibia requiring surgical intervention and who consented to avail teleconsultation services were included. All these patients were assessed by patient satisfaction score pre-operatively. Patients were randomly assigned for post-operative care schedules of 5 and 14 days in telemedicine and inpatient visits during their follow-up period.

Results: A total of 50 patients were included and the satisfaction rating among the two groups was identical. The average patient satisfaction score (on a 10-point scale) was found to be 9.77 in the inpatient visits based on the one-on-one follow-up group and 9.79 in the telemedicine consultation group.

Conclusion: In orthopedics, the implementation of telemedicine can minimize the need for the patient to physically visit the outpatient department. The rates of response and overall patient satisfaction were high in the telemedicine group. However, more efforts should be made to address the limitations and problems of using telemedicine.

## Introduction

Over the past several years, multiple forms of telemedicine have enabled healthcare personnel to improve healthcare delivery at a distance by monitoring post-operative follow-ups and interpret diagnostic imaging. The western world is using telemedicine facilities more often than countries like India. Although owning to the coronavirus disease 2019 (COVID-19) pandemic, the healthcare system has been disrupted globally.

Therefore, in March 2020, Indian authorities (National Medical Council) formulated guidelines for the appropriate use of telemedicine by registered medical practitioners and surgical and medical specialists to reduce disruption in healthcare and prevent the further spread of COVID-19 [1]. The utilization of telemedicine technologies will be advantageous both for the patients as well as healthcare providers. This provides better treatment outcomes in orthopedic settings as it is a multifaceted surgical specialty [1,2]. To counter COVID-19 spread, many institutions in China and the United States reportedly increased their use of telemedicine in surgical specialties, and have recently issued recommendations for its execution in treating appropriate patients. However, due to the pandemic, it is difficult to gather sufficient feedback regarding patient and provider satisfaction with telemedicine. Moreover, in Indian literature, the data regarding the use of telemedicine in orthopedic practice are scarce; therefore, the present study was designed with a goal to compare post-operative patient satisfaction while using telemedicine and in-person hospital visits of patients during the COVID-19 period [3,4].

## Materials and methods

Study design

The present one-year (May 2020 to April 2021) prospective observational study was conducted in the Department of Orthopedics, Government Medical College and Hospital, Patiala, Punjab, India. The study has been approved by the Government Medical College, Patiala with the registration number TRG.9(310)2020-35217.

Patient selection

All the patients who presented with fractures of the upper end of the tibia and requiring surgical intervention were evaluated. After an extensive preoperative evaluation (which included routine investigations like COVID-19 reverse transcription-polymerase chain reaction [RT-PCR], blood counts, biochemical investigations, and radiological tests), patients who consented to the study were included.

Inclusion criteria include patients with fresh fractures of the upper end of the tibia and age between 18 and 60 years.

Exclusion criteria used were patients with pathological fractures, fractures with impending compartment syndrome, compound fractures, and comminuted fractures, patients with co-morbid conditions (hypertension, diabetes mellitus, and chronic liver/kidney/cardiopulmonary disease), patients less than 18 years and above 60 years, and patients who did not consent to enrollment in the study.

Pre-operatively, all these patients were assessed by patient satisfaction score. Further, the patients were randomly assigned for post-operative care schedules of 5 and 14 days in telemedicine and inpatient visits during their follow-up period. Telemedicine patients used their gadgets like computers or mobile devices through telemedicine programs.

Outcome measurement

Both the groups were evaluated using validated pain scores and patient satisfaction scores.

Telemedicine consultations were given to the patients as per the national guidelines and were given without any charges during the study period. Patients who had telephonic consultations were asked to fill up a questionnaire a week after telephonic consultations. The pain scale of patients was measured with a numerical scale ranging from 0 to 10, with 0 indicating no pain, 1 indicating mild pain, 4-6 indicating minor pain, and 7-10 indicating severe pain.

The satisfaction score was calculated using an 11-point ordinal scale with five anchoring points. Patients gave feedback using the following scores from 0 to 10: (0-1) this visit was not what I hoped; (2-4) it could be better in many ways; (5) visit was helpful; (6-8) exceptionally beneficial visit; and (9-10) one of the most beneficial visits [5].

## Results

A total of 50 patients participated in the study with a majority (80%) of male patients (M:F ratio = 4:1). Majority of the patients were in 46-60 years age group (n = 22; 44%) followed by 31-45 years age group (n = 21; 42%) and 18-30 years age group (n = 7; 14%). Majority (30%) of the patients had type 4 fracture followed by type 3 (20%) and type 5 (20%), and type 1 (4%) being the least common type (Tables 1-3).

**Table 1:** Gender distribution of all patients OPD, outpatient department.

Sex	Mode of follow-up				Total	
	OPD		Telemedicine			
	No.	%	No.	%	No.	%
Female	5	20%	5	20%	10	20%
Male	20	80%	20	80%	40	80%
Total	25	100%	25	100%	50	100%

**Table 2:** Age distribution of all patients OPD, outpatient department.

Age group (years)	Mode of follow-up				Total	
	OPD		Telemedicine			
	No.	%	No.	%	No.	%
18-30	4	16%	3	12%	7	14%
31-45	10	40%	11	44%	21	42%
46-60	11	44%	11	44%	22	44%
Total	25	100%	25	100%	50	100%

**Table 3:** Fracture classification of all patients OPD, outpatient department.

Fracture Type	Mode of follow-up				Total	
	OPD		Telemedicine			
	No.	%	No.	%	No.	%
Type 1	1	4%	1	4%	2	4%
Type 2	4	16%	4	16%	8	16%
Type 3	5	20%	5	20%	10	20%
Type 4	7	28%	8	32%	15	30%
Type 5	5	20%	5	20%	10	20%
Type 6	3	12%	2	8%	5	10%
Total	25	100%	25	100%	50	100%

During the immediate postoperative period, patient satisfaction was equivalent in both categories. In both these groups, the surgeon enquired about surgical findings, post-operative recovery duration, degree, and duration of pain, and physical examinations, which included motion range testing, any discharge from the operative site, fever, and redness around the wound. During telemedicine follow-up, the surgeons did not touch the knee physically but examined the patient virtually. The assessment of wound healing, discharge, and swelling was done using visual aid.

In this study, satisfaction ratings among the two groups were found to be identical. The average patient satisfaction score (on the scale of 0-10) was found to be 9.77 and 9.79 in the inpatient visit follow-up and telemedicine follow-up, respectively. During this study, some of the patients (about 15%) have preferred other alternative types of follow-up. Analyzing pain scores showed similar improvement in both groups on the day of surgery and at follow-up (Tables 4, 5).

**Table 4:** Preoperative pain score and patient satisfaction score OPD, outpatient department.

Preoperative pain score	Mode of follow-up				Total	
	OPD		Telemedicine			
	No.	%	No.	%	No.	%
Mild	-	-	-	-	-	-
Moderate	-	-	-	-	-	-
Severe	25	100%	25	100%	50	100%
Total	25	100%	25	100%	50	100%
Preoperative satisfaction score	Mode of follow-up				Total	
	OPD		Telemedicine			
	No.	%	No.	%	No.	%
Satisfactory	-	-	-	-	-	-
Dissatisfactory	-	-	-	-	-	-
Neutral	-	-	-	-	-	-
Satisfactory	-	-	-	-	-	-
Very satisfactory	25	100%	25	100%	50	100%
Total	25	100%	25	100%	50	100%

**Table 5:** Postoperative pain score and patient satisfaction score OPD, outpatient department.

Postoperative pain score		Mode of follow-up						Total	
		OPD				Telemedicine			
		No.		%		No.	%	No.	%
Mild		17		68%		16	64%	33	66%
Moderate		8		32%		9	36%	17	34%
Severe		-		-		-	-	-	-
Total		25		100%		25	100%	50	100%
Postoperative satisfaction score	Mode of follow-up							Total	
	OPD				Telemedicine				
	No.		%		No.		%	No.	%
Very unsatisfied	-		-		-		-	-	-
Unsatisfied	-		-		-		-	-	-
Neutral	-		-		-		-	-	-
Satisfied	-		-		-		-	-	-
Very satisfied	25		100%		25		100%	50	100%
Total	25		100%		25		100%	50	100%

There were low complication rates among these groups. Three patients in the outpatient department (OPD) follow-up had shown pain and swelling and two patients in telemedicine had shown pain and swelling. These findings of patients had shown doubt of possible blood clots or infection-related complications. All these patients were investigated on the same days with tests like blood parameters and Doppler ultrasound scans of concerned parts, which showed no significant evidence of venous thromboembolism or infection. Patients had been assessed for any missed or subsequent complications.

## Discussion

Due to the emergence of COVID-19, the aim of the entire health system is to reduce and prevent the spread, morbidity, and mortality due to COVID-19. This has led to the diversion of medical resources, thus compromising on the routine outpatient department services and therefore limiting the routine medical care, especially by in-person visits. To encounter this major problem, many hospitals throughout the world opted for telemedicine services. Even in developing countries like India, the government issued guidelines for telemedicine services. But lack of awareness and scarcity of literature make telemedicine practice more challenging for both healthcare providers and patients [6-8].

Loeb et al. also examined experience regarding the introduction of telemedicine during the COVID-19 crisis, where the authors estimated that 50% of the clinical volume was maintained with telemedicine services provided by their hospital. Similarly in the present study, in both groups, the surgeon enquired about surgical findings, post-operative recovery period, degree of pain, and physical examination, which included the motion range testing, any discharge from the operative site, fever, and redness around the wound. During telemedicine follow-up, the surgeon did not feel or even touch the knee, but the surgeon could analyze patients by visual assessment of wound healing, discharge, and swelling around the knee [9-12].

Buvik et al. stated that visual consultations are less impactful when compared to normal consultations, and no significant difference in the number of surgically treated patients was detected across groups (p = 0.60) [13]. In an orthopedic randomized controlled trial, Haukipuro et al. discovered that the satisfaction rate of patients was equivalent in the telemedicine and conventional OPD follow-up group [14].

The advantages of orthopedic telemedicine, on the other hand, are not without significant drawbacks, which are linked to a lack of awareness of patients regarding telemedicine choices. As per a survey by J.D. Power (2019), 29% of patients who never used telemedicine stated about the unavailability of such services while 37% lacked awareness regarding telemedicine being given by their healthcare provider [15]. Also, orthopedic surgeons have apprehensions regarding the inability to perform physical examinations, for example, numerous maneuvers like manual motor testing, sensory examination, reflex testing, etc. done during physical examination are impossible to perform via virtual consultations [16]. Moreover, a 2017 survey by the American Orthopaedic Association, revealed that only 20% of orthopedic surgeons had faith in the usefulness of telemedicine for postoperative follow-up whereas 42% suggested that majority of their fellows would be disinterested in providing telephonic consultations [17].

In this study, the satisfaction rating among the two groups was statistically insignificant. Mean patient satisfaction scores (on a 10-point scale) were 9.77 in the inpatients visit-based follow-up and 9.79 in telemedicine follow-up. In the present study of post-operative patients, the satisfaction score was 100%, which was comparable to other studies done focused on orthopedics patients.

Couturier et al. (1998) and Aarnio et al. (1999) used video-conference (n = 15) and reported 80% and 87% satisfaction rate, respectively [18,19]. Williams et al. (2008) from the United Kingdom used telephone services (n = 598) and had a higher satisfaction rate of 93% [20]. More recently, Buvik et al. (2019) from Norway (n = 199) and Coronado et al. (2020) from the USA reported satisfaction rates of 99% and 100%, respectively [13,21]. The trends signify that the satisfaction rates have increased over the years probably because of the better acceptability of technologies available among both patients and healthcare providers. Similarly, Kumar et al. (2020) from India who used telephone services (n = 450) had 92% of patients satisfied with telemedicine thus emphasizing that telemedicine is being accepted in orthopedic practice in India as well [8].

The role of telemedicine in postoperative care has been emphasized again and again in western literature. Marsh et al. compared one-on-one consultations and postoperative follow-up (at least one year) by telemedicine in 229 patients (118 in telemedicine and 111 in the usual-care group) and suggested telemedicine being a more economical and time-saving option [22]. In addition, Thomas et al. illustrated the essentiality of telemedicine as a method for preoperative education regarding arthroplasty patients and thus helping in better postoperative rehabilitation [23]. Eichler et al., in a randomized controlled study, demonstrated better functional outcomes, reduced pain, and enhanced life quality. Similarly, the present study attempts to evaluate pain score and patient satisfaction score both preoperatively and postoperatively [24].

Limitations

The present study is a preliminary observation study and is limited by the small sample size and detailed questionnaire-based follow-up and lack of knowledge of the educational status of the participants. However, it can be considered as a progressive step toward improving patient outcomes using telemedicine, and therefore larger studies with large sample sizes and longer follow-ups can be carried out for better understanding.

## Conclusions

The improvement and utilization of telemedicine have increased as an impact of the COVID-19 pandemic, which has caused a sudden shift toward the utilization of virtual orthopedic assessments as an important measure. Therefore, with eased guidelines, better cost-effectiveness, and increased awareness among orthopedic surgeons, healthcare providers and patients can improve the quality and efficiency of virtual visits.
